# Association of Olfactory Dysfunction, Subjective Cognitive Decline and Alzheimer’s Biomarkers With Dementia Risk

**DOI:** 10.1093/geroni/igaf122.1082

**Published:** 2025-12-31

**Authors:** Mattie Derivaux, Xiaoqian Zhu, Srishti Shrestha, Kevin Sullivan, Michael Griswold, B Gwen Windham, Priya Palta, Vidyulata Kamath

**Affiliations:** University of Mississippi Medical Center, Jackson, Mississippi, United States; University of Mississippi Medical Center - The MIND Center, Jackson, Mississippi, United States; University of Mississippi Medical Center - The MIND Center, Jackson, Mississippi, United States; University of Mississippi Medical Center - The MIND Center, Jackson, Mississippi, United States; University of Mississippi Medical Center - The MIND Center, Jackson, Mississippi, United States; University of Mississippi Medical Center - The MIND Center, Jackson, Mississippi, United States; UNC School of Medicine, Chapel Hill, North Carolina, United States; Johns Hopkins University School of Medicine, Baltimore, Maryland, United States

## Abstract

Integrating early dementia manifestations, including subjective cognitive decline (SCD, self-reported cognitive changes without objective evidence) and olfactory dysfunction (OD), with plasma biomarkers of Alzheimer’s and neurodegeneration (Amyloid-β (Aβ) 42/40, phosphorylated-tau-181, Glial Fibrillary Acidic Protein, and Neurofilament Light Chain) may enhance early dementia risk stratification. Utilizing ARIC study data, we examined the combined associations of SCD, OD, and plasma biomarkers with incident dementia. We included 4,641 dementia-free participants (age=75±5y; 21% Black, 11% OD, 39% SCD, 21% mild cognitive impairment (MCI)) at ARIC Visit 5 (2011-2013) with complete information on OD (12-item Sniffin’ Sticks score ≤6) and cognition (categorized as SCD, normal cognition without SCD, or MCI based on subjective reports and objective cognitive adjudication). 1,430 participants also had plasma biomarker data at Visit 5 (categorized using median cut-offs). Incident dementia was identified via expert-adjudicated cognitive evaluation and hospital discharge or death records through December 31, 2022 (median follow-up=8y). Hazard ratios (HRs) were estimated using Cox regression, adjusting for demographic and cardiovascular factors. Model 1 (olfaction/cognition) showed participants with OD/SCD had more than double the dementia risk (HR = 2.86 (95% CI:2.12–3.86)) than those with No OD/normal cognition, exceeding risks for those with OD/normal cognition (HR = 1.99 (CI:1.45–2.74)) and No OD/SCD (HR = 1.45 (CI:1.20–1.75)). Model 2 (olfaction/cognition/amyloid pathology) demonstrated that participants with OD/SCD/worse amyloid pathology (Aβ42/40

